# Smart contracts software metrics: A first study

**DOI:** 10.1371/journal.pone.0281043

**Published:** 2023-04-12

**Authors:** Roberto Tonelli, Giuseppe Antonio Pierro, Marco Ortu, Giuseppe Destefanis

**Affiliations:** 1 Dept. of Computer Science and Mathematics, University Of Cagliari, Cagliari, Italy; 2 Dept. of Economics and Business Sciences, University Of Cagliari, Cagliari, Italy; 3 Dept. of Computer Science, Brunel University, Uxbridge, London, United Kingdom; Hanyang University, KOREA, REPUBLIC OF

## Abstract

Smart contracts (SC) are software programs that reside and run over a blockchain. The code can be written in different languages with the common purpose of implementing various kinds of transactions onto the hosting blockchain. They are ruled by the blockchain infrastructure with the intent to automatically implement the typical conditions of traditional contracts. Programs must satisfy context-dependent constraints which are quite different from traditional software code. In particular, since the bytecode is uploaded in the hosting blockchain, the size, computational resources, interaction between different parts of the program are all limited. This is true even if the specific programming languages implement more or less the same constructs as that of traditional languages: there is not the same freedom as in normal software development. The working hypothesis used in this article is that Smart Contract specific constraints should be captured by specific software metrics (that may differ from traditional software metrics). We tested this hypothesis on 85K Smart Contracts written in Solidity and uploaded on the Ethereum blockchain. We analyzed Smart Contracts from two repositories “Etherscan” and “Smart Corpus” and we computed the statistics of a set of software metrics related to Smart Contracts and compared them to the metrics extracted from more traditional software projects. Our results show that generally, Smart Contract metrics have more restricted ranges than the corresponding metrics in traditional software systems. Some of the stylized facts, like power law in the tail of the distribution of some metrics, are only approximate but the lines of code follow a log-normal distribution which reminds us of the same behaviour already found in traditional software systems.

## 1 Introduction

Smart Contracts have gained tremendous popularity in the past few years, to the point that billions of US Dollars are currently exchanged every day using such a technology. However, since the release of the Ethereum platform in 2015, there have been many cases in which the execution of Smart Contracts managing Ether coins led to problems or conflicts. Smart Contracts rely on a non-standard software life-cycle, according to which, for instance, delivered applications can hardly be updated or bugs resolved by releasing a new version of the software. Furthermore, their code must satisfy constraints typical of the domain such as the following:

- they must be light. Smart Contact definitions are limited in size because of structural constraints imposed by the Blockchain infrastructure and the mining cost;
- Smart Contract execution has a per operation cost so their execution must be limited;
- once published Smart Contracts are immutable: indeed a blockchain is based on the append-only mechanism—then code under the form of bytecode is inserted into a blockchain block once and forever [1];
- floating point values cannot be used due to the consensus among all the nodes on the blockchain status which contrasts with the possibility of different rounded values of floating point numbers on machines with different precision;
- random number generators cannot be used for the same reason and in their place hashing functions are commonly used.

The idea of Smart Contracts was originally described by cryptographer Nick Szabo in 1997, as a kind of digital vending machine [2].

*Smart contracts* are self-applying agreements, or contracts, implemented through a computer program whose execution enforces the terms of the contract. The idea is to remove a central supervisory authority, entity or organization that both parties must trust and delegate that role to the *correct* execution of a computer program. Such a scheme can therefore count on a decentralized system managed automatically by computers, and Blockchain technology is the tool to deliver the trust model envisaged by smart contracts.

Since smart contracts are stored on a blockchain, they are public and transparent, immutable and decentralised, and since blockchain resources are costly, their code size cannot exceed domain-specific constraints. Immutability means that when a smart contract is created, it cannot be changed again.

Smart contracts can be applied to many different scenarios: banks could use them to issue loans or to offer automatic payments; insurance companies could use them to automatically process claims according to agreed terms; postal companies could use them for payments on delivery. In the following, we mainly refer to the Ethereum technology without losing generality.

A *Smart Contract* (SC) is a *full-fledged program* stored in a blockchain by a *contract-creation* transaction. A SC is identified by a *contract address* generated upon a success creation transaction. A blockchain state is therefore a mapping from addresses to accounts. Each SC account holds an *amount of virtual coins* (Ether in our case), and has its own private *state* and *storage*.

**Fig 1:**
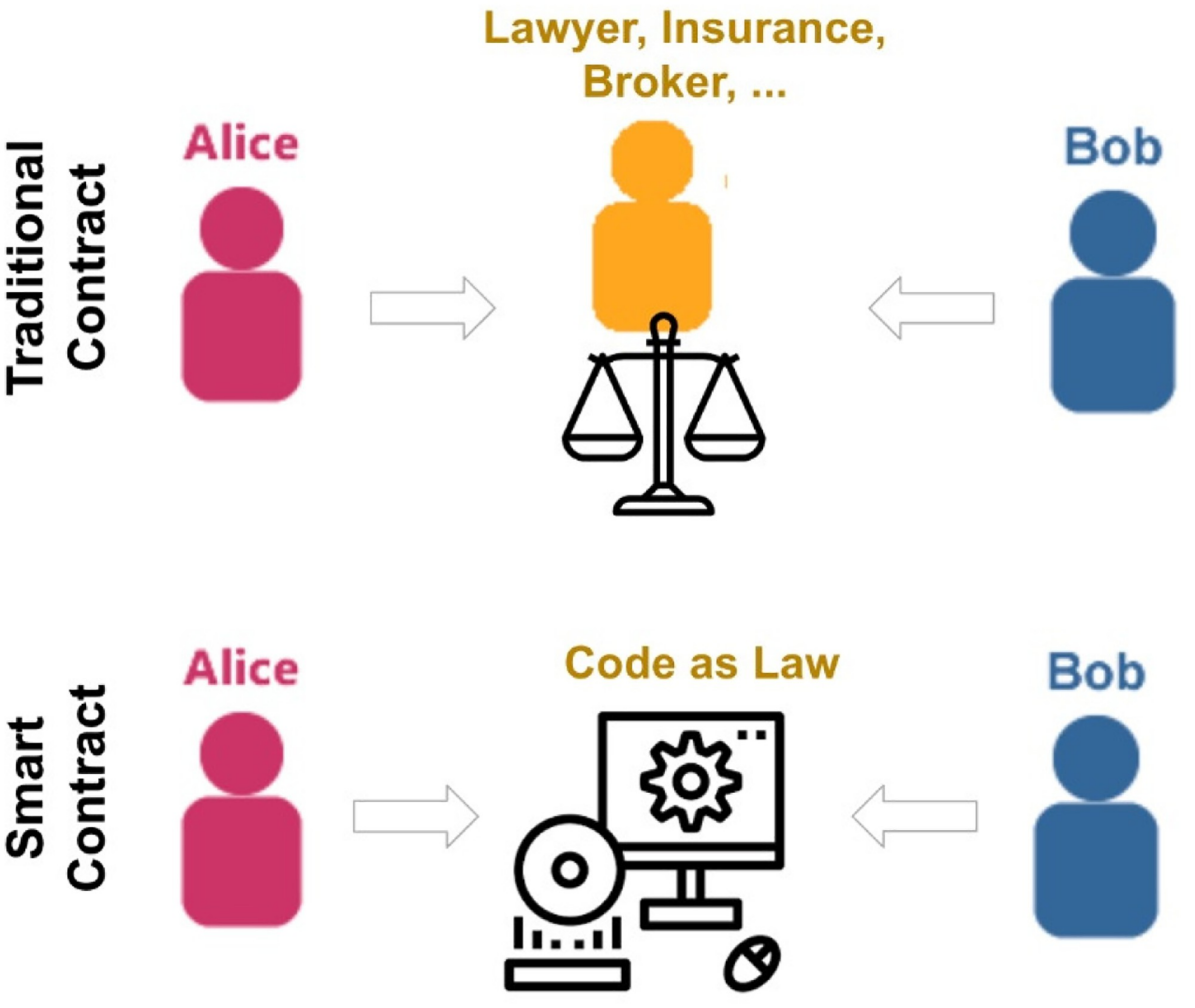
Smart contract vs. traditional contract.

Fig 1 illustrates how smart contracts work by comparing smart contracts to traditional contracts. “Smart contracts” differ from traditional contracts in that they are computer programs that automate certain aspects of an agreement between two parties through the use of blockchain technology. Indeed, blockchains provide security, permanence, and immutability through the replication of the smart contract code across multiple nodes.

The most used SC programming language is Solidity which runs on the Ethereum Virtual Machine (EVM) on the Ethereum blockchain. Since this is currently the most popular paradigm, we focus our attention on Solidity. An Ethereum SC account hence typically holds its executable code and a state consisting of:

- a private storage
- the amount of virtual coins (Ether) it holds, i.e. the contract *balance*.

Users can transfer Ether coins using transactions, like in Bitcoin, and additionally can *invoke* contracts using *contract-invoking* transactions. Conceptually, Ethereum can be viewed as a huge *transaction-based state machine*, where its state is updated after every transaction and stored in the blockchain.

Smart Contracts source code manipulate variables in the same way as traditional imperative programs. At the lowest level the code of an Ethereum SC is a stack-based bytecode language run by an Ethereum virtual machine (EVM) in each node. SC developers define contracts using high-level programming languages. One such language for Ethereum is Solidity [3] (a JavaScript-like language), which is compiled into EVM bytecode. Once a SC is created at an address *X*, it is possible to invoke it by sending a contract-invoking transaction to the address *X*. A contract-invoking transaction typically includes:

- payment (to the contract) for the execution (in Ether).
- input data for the invocation.

### 1.1 Working example

**Fig 2:**
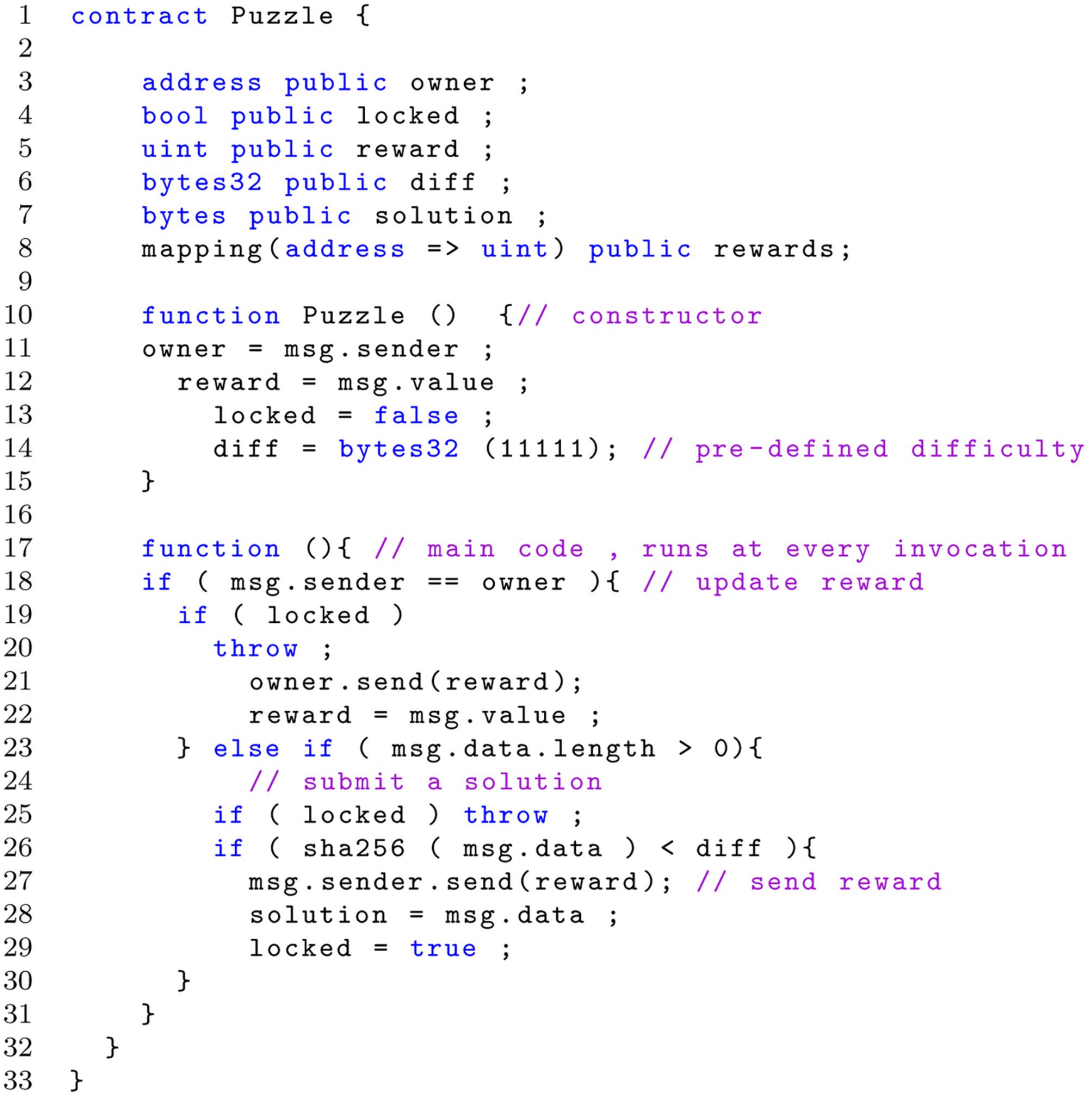
Smart contracts example.

Fig 2 shows a simple example of SC reported in [4], which rewards anyone who solves a problem and submit the solution to the SC. This contract has been selected as an example of an old style solidity smart contracts, in fact many of the constructs it uses are now deprecated, but it is instructive since it also represents how the solidity language and the metrics used in it changed along time.

A *contract-creation* transaction containing the EVM bytecode for the contract in Fig 2 is sent to miners. Eventually, the transaction will be accepted in a block, and all miners will update their local copy of the blockchain: first a *unique* address for the contract is generated in the block, then each miner executes locally the *constructor* of the **Puzzle** contract, and a local storage is allocated in the blockchain. Finally the EVM bytecode of the anonymous function of **Puzzle** (Lines 16+) is added to the storage.

When a *contract-invoking* transaction is sent to the address of **Puzzle**, the function defined at Line 16 is executed by default. All information about the sender, the amount of Ether sent to the contract, and the input data of the invoking transaction are stored in a default input variable called *msg*. In this example, the *owner* (namely the user that created the contract) can update the *reward* (Line 21) by sending Ether coins stored in msg.value (if statement at Line 17), after sending back the current *reward* to the *owner* (Line 20).

In the same way, any other user can submit a solution to **Puzzle** by a *contract-invoking* transaction with a *payload* (i.e., msg.data) to claim the reward (Lines 22-29). When a correct solution is submitted, the contract sends the reward to the sender (Line 26).

### 1.2 Gas system

It is worth remarking that a Smart Contract is run on the blockchain by each miner deterministically replicating the execution of the Smart Contract’s bytecode on the local copy of the blockchain. This, for instance, implies that to guarantee coherence across the copies of the blockchain, code must be executed in a strictly deterministic way (and therefore, for instance, the generation of random numbers may be problematic).

Solidity, and in general high-level Smart Contract’s languages, are Turing complete in Ethereum. Note that in a decentralised blockchain architecture Turing completeness may be problematic, e.g., the replicated execution of infinite loops may potentially *freeze* the whole network.

To ensure fair compensation for expended computation efforts and limit the use of resources, Ethereum pays miners some fees, proportionally to the required computation. Specifically, each instruction in the Ethereum bytecode requires a pre-specified amount of *gas* (paid in Ether coins). When users send a *contract-invoking* transaction, they must specify the amount of gas provided for the execution, called *gasLimit*, as well as the price for each gas unit called *gasPrice*. A miner who includes the transaction in his proposed block receives the transaction fee corresponding to the amount of gas that the execution has actually burned, multiplied by *gasPrice*. If some execution requires more gas than *gasLimit*, the execution terminates with an exception, and the state is rolled back to the initial state of the execution. In this case the user pays all the *gasLimit* to the miner as a counter-measure against resource-exhausting attacks [5].

The code in Fig 2 displays typical features of the Solidity Smart Contract’s code: the *Contract* declaration, addresses declarations and mapping, owner data managing and the functions with the specific code for implementing the contract and transactions between blockchain addresses. Most of the control structures from JavaScript are available in Solidity except for switch and goto. So there is: if, else, while, do, for, break, continue, return [6], with the usual semantics known from C or JavaScript.

Functions of the current contract can be called directly (Internal Function Calls), also recursively. These function calls are translated into simple jumps inside the EVM. This has the effect that the current memory is not cleared, i.e., passing memory references to internally-called functions is very efficient. Only functions of the same contract can be called internally. The expressions this.g(); and c.g(); (where c is a contract instance) are also valid function calls, but this time, the function will be called as External Function Call, via a message call and not directly via jumps. Functions of other contracts have to be called externally. For an external call, all function arguments have to be copied to memory. When calling functions of other contracts, the amount of cryptocurrency (Wei) sent with the call and the gas can be specified with special options .value() and .gas() respectively. Inheritance between contracts is also supported.

Since Smart Contracts are closely related to classes of object-oriented programming languages, it is straightforward to define and compute some of the software metrics typically encountered in object-oriented software systems, like number of lines of code, comments, number of methods or functions, cyclomatic complexity and so on, while it is somehow more difficult to recognize software metrics related to communication between smart contracts, since these can be ruled by blockchain transactions among contracts, which can act somehow as code libraries.

On the other hand smart contracts are deployed and work on the blockchain infrastructure and it is thus likely that typical value of the same metrics can differ from the typical values of the same metrics in traditional software systems.

It became thus interesting, even from a software engineering point of view, to perform a statistical analysis of Smart Contract software metrics and to compare the data with those displayed by traditional software systems. It would also be of primary interest to examine the connection between software metrics and software quality, a field of research well established in traditional software, in the specific domain of smart contracts given that it is well known that Smart Contract code vulnerability have been exploited to stole value in cryptocurrencies from smart contracts [3, 5, 7, 8].

In this paper, we perform the analysis on a data set of 85K smart contracts downloaded from 1) etherscan.io, a platform allowing enhanced browsing of Ethereum blockchain and smart contracts and 2) smart corpus [9], an organized smart contract repository.

Motivations for this study arise from the need to measure software artifacts in the specific case of Smart Contracts code. In fact there are no studies involving a full statistical analysis of the metrics properties for such software artifacts in the new paradigm of blockchain systems. Knowledge of software metrics statistical properties is fundamental for controlling software production process, software quality as well as to perform fault prediction and to identify code smells.

We collected the blockchain addresses, the Solidity source code, the ABI and the bytecode of each contract and extracted a set of standard and SC-specific software metrics such as number of lines of smart contract code (LOCs), line of comments, blank lines, number of functions, cyclomatic complexity, number of events calls, number of mappings to addresses, number of payable, number of modifiable and so on. We analyzed the statistical distributions underlying such metrics to discover if they exhibit the same statistical properties typical of standard software systems [10–12] or if the SM constraints act so that a sensible variation in these distributions can be detected. Furthermore, we devise a path to the analysis of which and to what extent the SC metrics influence Smart Contract’s performance, usage in the blockchain, vulnerabilities, and possible other factors related to the specific contracts which can be reflected on the domain of application for which the smart contract has been deployed, like, for example, to implement and rule an initial coin offer (ICO), to control a chain of certification like in medical applications and so on.

## 2 Related work

Blockchain technology and Smart Contracts rose an exponentially increasing interest in the last years in different fields of research. Organizations such as banking and financial institutions, and public and regulatory bodies, started to explicitly talk of the importance of these new technologies. Software Engineering specific for blockchain applications and Smart Contract is still in its infancy [13] and in particular the investigation of the relationships among Smart Contracts Software Metrics (SCSM) and code quality, SC performances, vulnerability, maintainability and other software features is completely lacking. Smart Contracts and blockchain have been discussed in many textbooks [14] and documents over the internet, where white papers usually cover the specific topic of interest [15–19].

Ethereum defines a smart contract as a transaction protocol that executes the terms of a contract or group of contracts on a cryptographic blockchain [20]. Smart Contracts operate autonomously with no entity controlling the majority of its tokens, and its data and records of operation must be cryptographically stored in a public, decentralized blockchain [14].

Smart Contract vulnerabilities have been analyzed in [21–23]. A taxonomy of Smart Contract is performed in [22], where Smart Contracts are classified according to their purpose. These are divided into wallets, financial, notary, game, and library.

Authors in [4] investigate the security of running smart contracts based on Ethereum in an open distributed network like those of cryptocurrencies and introduce several new security problems in which an adversary can manipulate smart contract execution to gain profit.

Obviously Smart Contract scientific literature is limited due to their recent creation. On the other hand there is a plethora of results and information to rely on produced in the last decades for what concerns the relationship among software metrics and software quality, maintainability, reliability, performance defectiveness and so on.

Measuring software to get information about its properties and quality is one of the main issues in modern software engineering.

Limiting ourselves to object-oriented (OO) software, one of the first works dealing with this problem is the one by Chidamber and Kemerer (CK), who introduced the popular CK metrics suite for OO software systems [24]. In fact, different empirical studies showed significant correlations between some of CK metrics and bug-proneness [24–28]. Metrics have been defined also on software graphs and were found most correlated to software quality [29–32]. Tosun et al. applied Social Networks Analysis to OO software metrics source code to assess defect prediction performance of these metrics [33]

The CK [34] suite is historically the most adopted and validated to analyze bug-proneness of software systems [24, 27].

CK suite was adopted by practitioners [24] and is also incorporated into several industrial software development tools. Based on the study of eight medium-sized systems developed by students, Basili et al. [25] were among the first to find that Object-Oriented metrics are correlated to defect density. Considering industry data from software developed in C++ and Java, Subramanyam and Krishnan [26] showed that CK metrics are significantly associated with defects. Among others, Gyimóthy et al. [27], studying a Open Source system, validated the usefulness of these metrics for fault-proneness prediction.

CK metrics are intended to measure the degree of coupling and cohesion of classes in object-oriented software contexts. Statistical analysis has also been used in literature to detect typical features of complex software and to relate the statistical properties to software quality.

Recently, some researchers have started to study the field of software to find and study associated power-law distributions. In fact, many software systems have reached such a huge dimension that it looks sensible to treat them using the stochastic random graph approach [35].

Examples of these properties are the lines of code of a class, a function or a method; the number of times a function or a method is called in the system; the number of time a given name is given to a method or a variable, and so on.

Some authors already found significant power-laws in software systems. Cai and Yin [11] found that the degree distribution of software execution processes may follow a power-law or display small-world effects. Potanin et al. [36] showed that the graphs formed by run-time objects, and by the references between them in object-oriented applications, are characterized by a power-law tail in the distribution of node degrees. Valverde et al. [37, 38] found similar properties studying the graph formed by the classes and their relationships in large object-oriented projects. They found that software systems are highly heterogeneous small world networks with scale-free distributions of the connection degree. Wheeldon and Counsell [12] identified twelve power laws in object-oriented class relationships of Java programs. In particular, they analyzed the distribution of class references, methods, constructors, field and interfaces in classes, and the distribution of method parameters and return types. Myers [39] found similar results on large C and C++ open source systems, considering the collaborative diagrams of the modules within procedural projects and of the classes within the Object-oriented projects. He also computed the correlation between some metrics concerning software size and graph topological measures, revealing that nodes with large output degree tend to evolve more rapidly than nodes with large input degree. Other authors found power-laws studying C/C++ source code files, where graph edges are the files, while the “include” relationships between them are the links [40, 41]. Tamai and Nakatani [42], proposed a statistical model to analyze and explain the distributions found for the number of methods per class, and for the lines of code per method, in a large object-oriented system.

While most of these studies are based on static languages, such like C++ and Java, Marchesi et al. [43] provide evidence that a similar behavior is displayed also by dynamic languages such as Smalltalk. Concas et al. found power-law and log-normal distributions in some properties of Smalltalk and Java software systems—the number of times a name is given to a variable or a method, the number of calls to methods with the same name, the number of immediate subclasses of a given class in five large object-oriented software system [10, 44]. The Pareto principle is used to describe how faults in large software systems are distributed over modules [45–49]. Baxter et al. [50] found power-law and Log-normal distributions in the class relationship in Java programs. They proposed a simple generative model that reproduces the features observed in real software graph degree distributions. Ichii et al. [51] investigated software component graphs composed of Java classes finding that in-degree distribution follows the power law distribution and the out-degree distribution does not follow the power-law. Louridas et al. [52], in a recent work, show that incoming and outgoing links distributions have in common long, fat tails at different levels of abstraction, in diverse systems and languages (C, Java, Perl and Ruby). They report the impact of their findings on several aspects of software engineering: reuse, quality assurance and optimization.

Given the vast literature investingating power law distributions in software systems, we choose to investigate these properties, also in SC software not only to look for power-law behaviour, but also because some features are related to design and coding guidelines, to software quality and also to Chidamber and Kemerer (CK) NOC metrics [24].

Wheeldon and Counsell [12], as well as other researchers, found power-laws in the distributions of many software properties, such as the number of fields, methods and constructors of classes, the number of interfaces implemented by classes, the number of subclasses of each class, as well as the number of classes referenced as field variables and the number of classes which contain references to classes as field variables. Thus, there is much evidence that power-laws are a general feature of software systems. Concas et al. [44] explained the underlying mechanism through a model based on a single Yule process in place during the software creation and evolution.

More recently affect metrics have been investigated revealing how during software development productivity and software quality can be highly influenced by developers moods [53–58].

In [59] authors review papers relating to smart contracts metrics and other five specific topics: smart contract testing, smart contract code analysis, smart contract security, Dapp performance, and blockchain applications.

A few studies investigated SC metrics and collected a curated repository of SC [9, 59–62].

In [63] authors examined SCs extracted from various Ethereum blockchain-oriented software projects hosted on GitHub.com, extracting also a suite of object-oriented metrics, to evaluate their structural characteristics.

More recently, deep learning neural networks have been used [64, 65] where to develop a deep learning framework for detecting fraudulent smart contracts on blockchain systems and hybrid deep learning models combining different word embedding methods, for smart contract vulnerability detection.

## 3 Experimental set-up

Etherscan [66] is a web based platform which allows for Ethereum blockchain exploration of all blockchain addresses. It allows one to recover Smart Contracts bytecode, ABI, and it collects also Smart Contract source codes in Solidity Part of the data used in this paper (15% of the total) have been retrieved by analyzed the blockchain addresses related to the available source code on Etherscan. These addresses have been used to systematically download the code of the Solidity contracts, as well as the bytecode and information associated with the ABI.

Smart contracts analyzed in this study can be found online through a tool named Smart Corpus [9]. Smart Corpus is a collection of over 100K smart contracts categorized by software metrics (number of lines of code, cyclomatic complexity, etc.) and uses cases (banks, finance, betting, hectares, etc.). A detailed description of the Smart Corpus tool and its related publication can be found here (https://aphd.github.io/smart-corpus/). After collected and locally stored Solidity code, bytecode, and ABI infos, we built a code parser to extract the software metrics of our interest for each smart contract. We also manually explored the code to get insights into the more relevant information to eventually extract from the data and to get a flavour of the main features of the overall dataset. This exploratory analysis allowed us to note how the same contract code is often replicated and deployed to different blockchain addresses or deployed with very little changes. This pattern reveals how many contracts are simply experiments or are deployed to the blockchain for testing and then modified according to test’s results. They usually appear in a series of neighbour blockchain blocks. The dataset has thus a little bias but the overall effect is negligible in our analysis since there are very few cases of replicated Solidity code.

The dataset source code has been then parsed for computing total lines of code associated to a specific blockchain address, the number of smart contracts inside a single address code (the analogous of classes into java files, e.g., compilation units), blank lines, comment lines, number of static calls to events, number of modifiers, number of functions, number of payable functions, cyclomatic complexity as the simplest McCabe definition [67], and number of mappings to addresses.

We also computed the size of the associated bytecode and of the vector of contract’s ABIs. These are the Application Binary Interfaces, defining the interface definition of any smart contract, known at compilation time and static. All contracts will have the interface definitions of any contracts they call available at compile-time [68]. This specification does not address contracts whose interface is dynamic or otherwise known only at run-time.

The data set is structured to keep track of the specific Smart Contract address so that any blockchain address related Smart Contract metrics (SCEM: smart contract external metrics) can be fully analyzed in relationship with the software metrics self-contained into the Smart Contract Solidity code (SCIM: smart contract internal metrics). For example, it is possible to investigate interactions with other Smart Contracts, gas consumption and cryptocurrency exchanges.

ABI metrics in particular are the Smart Contract interface and reflect the external exposure of the Smart Contract towards blockchain calls from other addresses, which can be interaction with other Smart Contracts as well.

It is worth noting that not all the measures related to addresses stay constant but many of them depend on the time of analysis and cannot be defined among the Smart Contract metrics, and others can simply be contract variables, like the amount of ether stored into the contract, the number of owners in a multi owned contract, the contract performance, or popularity in terms of calls to the contract. In such cases, much care is needed to evaluate the relationship between Smart Contract software metrics and other blockchain-related measures, not only because they may be time-varying, but also because other external factors can be in place. For example, the success of a contract could be defined in terms of calls to that contract, but if the contract implements an Initial Coin Offer, then most likely the contract in itself, measured as software code, has probably little to do with it.

For each software metric we computed standard statistics like average, median, maxima and minima values and standard deviation. Furthermore we verified what kind of statistical distribution these metrics belong to. This is particularly important when comparing Smart Contract’s source code with other source code metrics, e.g., Java source code, for standard software projects. In fact the literature on software metrics demonstrates that there exist statistical distributions which are typical of specific metrics regardless the programming language used for software development [69].

In particular LOC, coupling metrics, like fan-in and fan-out, and other software metrics are known to display a fat tail in their statistical distribution [52] regardless the programming language, the platform or the software paradigm adopted for a software project.

Due to the domain specific constraints the Smart Contract software must satisfy to, in particular limited size resources, it is not granted that such software metrics respect the canonical statistical distributions found in general purpose software projects. It is one of the aims of this research to verify and eventually discuss such a conjecture.

## 4 Results

The smart contracts’ source code was analysed with a tool named PASO. Thanks to this tool the smart contract’s source code can be represented as an abstract syntax tree (AST). Based on the AST, software metrics and patterns in smart contract codes have been evaluated and computed. Detailed information about this tool and its publication can be found online at this link (https://aphd.github.io/paso/).

We started analyzing centrality and dispersion measures for all the computed metrics, like mean, average, median, and standard deviation, interquartile range, and total variation range. These statistics provide a summary of the overall behavior for the metrics values. In particular, for asymmetric distributions, centrality measure differs from one another, and in the case of power laws, distributions the largest values of the metrics can be order of magnitude larger than central and low values.

Many minima values result set to zero, since there are a few contracts with almost no code. The results on central tendency measures in Table 1 show that the mean is constantly larger than the median, (almost always of about two third) which is a feature typical of right skewed distributions. One simple reason explaining this fact is the lower bound posed to all the metrics by the fact that they are defined null or positive, while in principle, large values are not bounded. A little exception is represented by the Bytecode metric which features values for mean and median very close to each other, suggesting a distribution shape which may be not really skewed. Standard deviations are all comparable with the mean, meaning a large dispersion of values around the last, but there are not cases where it is much large than the mean or the media. Values of standard deviation much larger than the mean might be instead the case for power law distributions and such behavior has already been observed in software metrics for typical software systems [12, 44].

**Table 1:** Centrality and dispersion statistics computed for all the Smart Contract software metrics.

variable	Mean	Median	Std	Min	Max	IQR	10th	90th
total_lines	586.96	317.00	937.23	1	25,920	525.00	93.00	1,373.00
blanks	91.69	54.00	160.31	0	4,045	77.00	13.00	201.00
functions	44.96	28.00	66.27	0	1,256	36.00	9.00	95.00
payable	2.00	1.00	6.40	0	205	2.00	0.00	5.00
events	5.08	3.00	6.08	0	137	4.00	1.00	11.00
mapping	4.11	3.00	4.67	0	155	2.00	0.00	8.00
modifiers	1.86	1.00	2.48	0	40	3.00	0.00	5.00
contracts	7.29	5.00	9.52	1	227	6.00	2.00	14.00
interfaces	1.28	0.00	2.55	0	52	1.00	0.00	5.00
libraries	1.22	1.00	1.87	0	36	2.00	0.00	3.00
addresses	55.27	36.00	91.31	0	2,500	40.00	9.00	108.00
cyclomatic	66.50	36.00	105.66	0	2,318	55.00	13.00	146.00
comments	72.77	38.00	198.16	0	25,536	68.00	1.00	154.00
abiLength	221.60	144.00	586.81	0	34,728	113.00	66.00	310.00
abiStringLength	4,644	3,886	3,282	2	48,274	3,030	1,671	8,375
bytecode	12,483	9,606	9,953	2	49,152	10,714	3,336	26,921
LOC	306.63	167.00	529.08	1	14,151	240.75	64.00	663.00
block	47.83	28.00	72.34	0	1,534	39.00	10.00	102.00
isFallback	0.38	0.00	0.55	0	8	1.00	0.00	1.00
isVirtual	4.70	0.00	17.98	0	462	0.00	0.00	18.00
pure	5.58	4.00	9.67	0	209	7.00	0.00	13.00
view	12.22	6.00	28.86	0	650	14.00	0.00	33.00

The maxima are all much larger than the corresponding means and medians, often reach one or two order of magnitude larger and only in a few cases three orders of magnitude. Finally the 90th percentiles are comparable with a displacement of some standard deviation from the mean. All these results suggest that the selected Smart Contracts metrics might not display fat tail or power law distributions which are instead found in the literature for corresponding metrics of standard software systems.

Nevertheless outlier values appear for all the metrics and the values in Table 1 are not exhaustive for explaining completely their statistical properties.

Table 2 shows the Solidity programming statements statistics computed for all the 85K Smart Contracts composing our dataset. Based on statements’ statistic, a typical Smart Contract consists of almost 10 IF’s statements, 5 EMIT’s statements and 1.5 iteration statements. The same overall distribution of statement types was obtained in different periods of time with varying versions of solidity. So the statistic tends to be relatively stable. Notably, the number of iteration statements per line of code (0.005) is two orders of magnitude smaller than other programming languages such as Java (0.121), C and python. The number of conditional statements per line of code (0.033) is one order of magnitude smaller than other programming languages such as Java (0.142), C and python. The third most used statement in Smart Contracts after the return statement and IF statement is the EMIT’s statement. The Emit statement is used to release an event in a Smart Contracts, which can be read by the client in a decentralized application (dApp).

**Table 2:** Statements statistics computed for all the Smart Contracts.

variable	Mean	Median	Std	Min	Max	IQR	10th	90th
ifStatement	9.97	3.00	23.04	0	621	10.00	0.00	22.00
doWhileStatement	0.00	0.00	0.09	0	7	0.00	0.00	0.00
emitStatement	4.93	4.00	6.96	0	130	7.00	0.00	11.00
whileStatement	0.33	0.00	1.11	0	24	0.00	0.00	1.00
forStatement	0.95	0.00	2.26	0	13	1.00	0.00	3.00
inlineAssemblyStatement	0.90	0.00	2.98	0	81	1.00	0.00	2.00
returnStatement	21.80	14.00	30.05	0	712	19.00	3.00	45.00
revertStatement	0.01	0.00	0.30	0	37	0.00	0.00	0.00
throwStatement	0.53	0.00	2.96	0	75	0.00	0.00	0.00
tryStatement	0.06	0.00	0.41	0	25	0.00	0.00	0.00

To perform a complete analysis, we proceed in two steps. We perform a first qualitative investigation analyzing the histograms for all the metrics, then we use more complex statistical models for best fitting the Empirical Complementary Cumulative Distribution Function to extract quantitative information on Smart Contracts software metrics. The histogram patterns are well known to depend on the bin size and number, as well as on the local density of points into the various ranges. Nevertheless they can be an helpful instrument to get insight into the distribution shape general features, namely if there may be fat tails, bulk initial distribution values and so on. On the contrary the best fittings functions with statistical models provide precise values of core parameters and can be compared with those reported in literature for standard software metrics.

**Fig 3:**
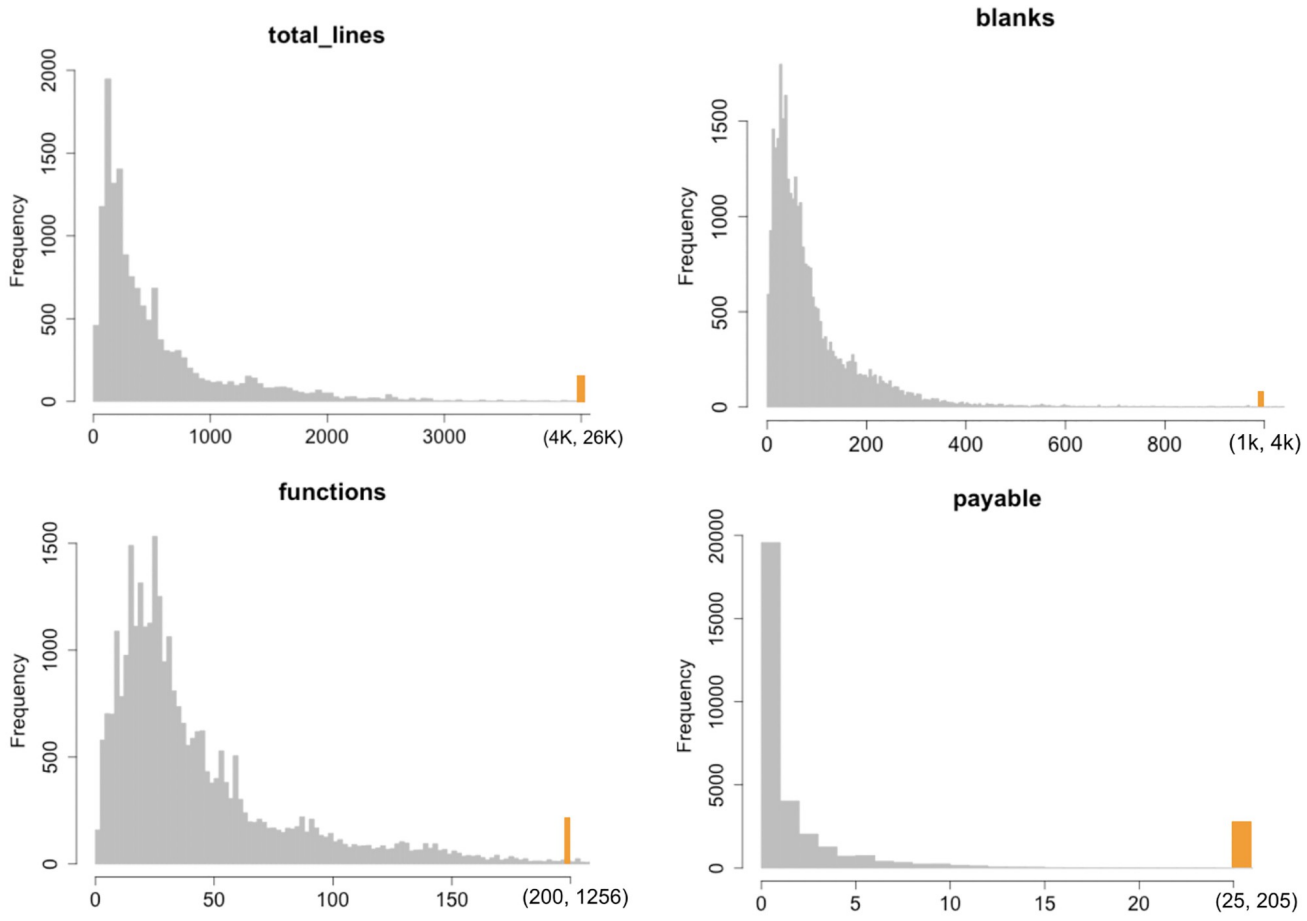
Histogram distributions of the metrics Total lines, Blanks, Function and Payable.

**Fig 4:**
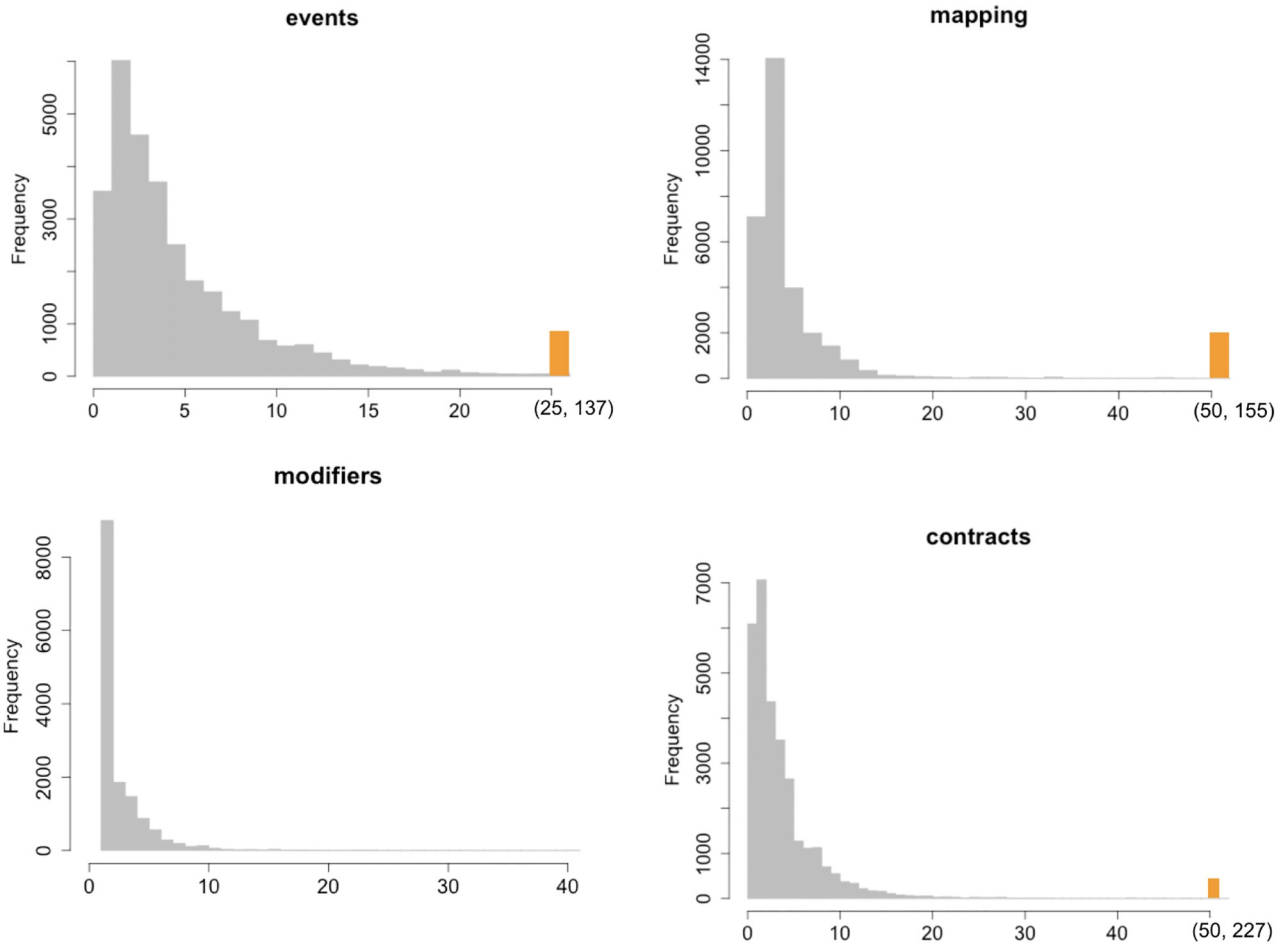
Histogram distributions of the metrics Events, Mapping, Modifier and Contract.

**Fig 5:**
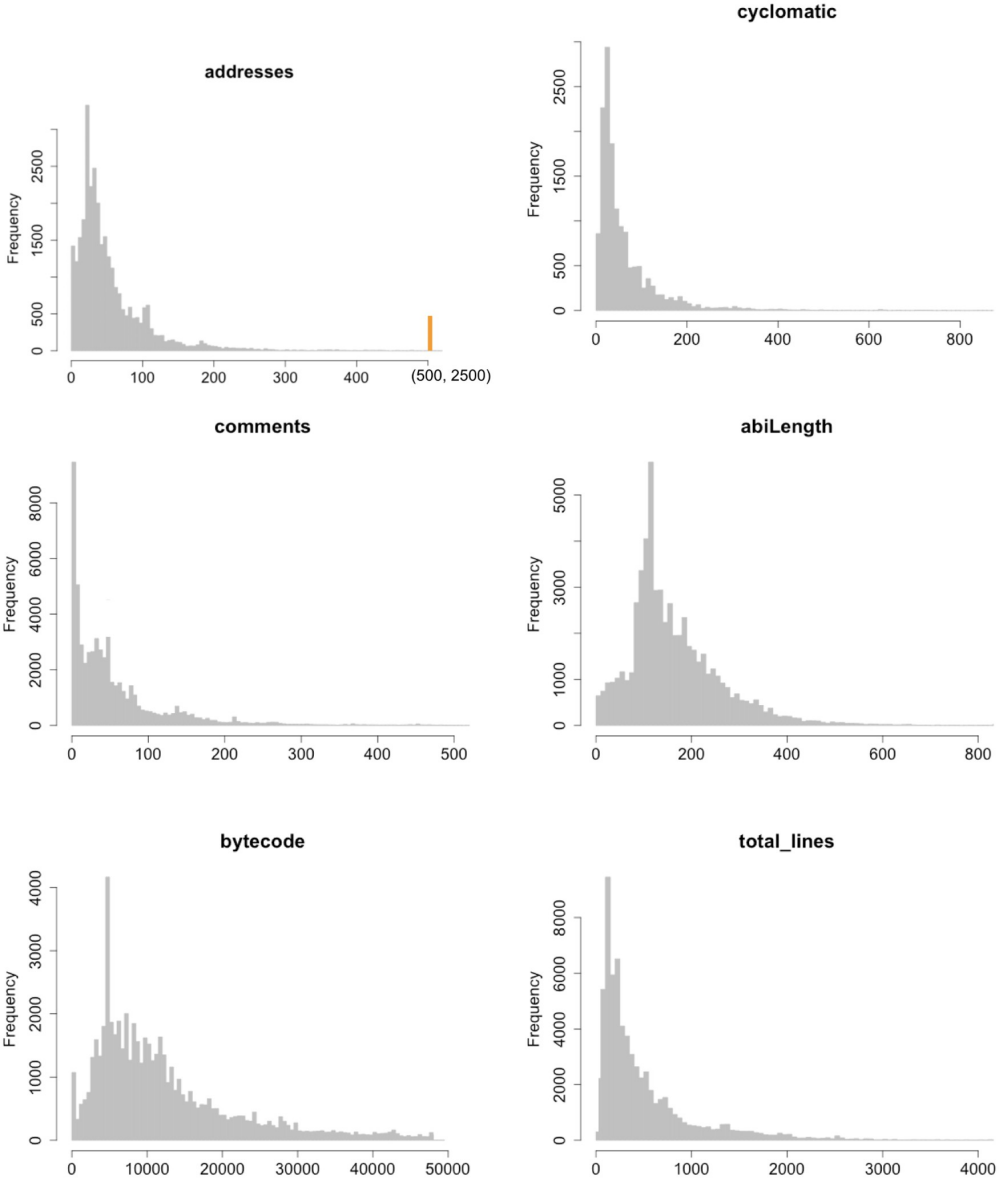
Histogram distributions of the metrics Address, Cyclomatic, Comments, ABI, Bytecode and LOCS.

In Figs 3–5 we report the histograms for all the Smart Contracts software metrics in the same order they are reported in Table 1. To make the histograms more readable, the range of the last bin is highlighted with a different fill colour. The orange-colored bin represents the outlier aggregation. The general shape can be distinguished into two categories. From one side there are those metrics whose ranges of variations are quite limited and maximum values are below 250, like Payable, Events, Mapping, Modifiable. For such metrics the histograms contain too few different values which does not allow to display a power law behavior. In particular Payable and Modifiable appear also to have a bell shape which allows to exclude a general power law distribution. For Events and Mapping the shape may suggest a power law behavior which is limited by the upper bounds reached by the maximum metric values. This deserves to be better investigated using statistical distribution modeling.

From the other side the metrics which reach values large enough (whose maxima are over 250) contain enough points to well populate the histograms. Also in this case many metrics have bell shaped distributions with limited asymmetry and skewness. This feature can be ascribed to the limited range of values these metrics can reach. In fact, in cases where the metrics can assume virtually arbitrary large values, many orders of magnitude larger that their mean values, the bell shape disappear and the shape presents a strong asymmetry with a high skewness. This is the behavior observed in literature for metrics in common software systems. The only cases where a full power law distribution may approximately hold are those related to the lines of code, like total lines of code, blank lines, comments and LOC. But also in these cases the upper bound of the values of the metrics does not allow to fully acknowledge for the power law. This seems to be a structural difference with respect to standard software systems where the number of lines of code for a class, for example in Java systems, may easily reach tens of thousands. In fact such systems rely on *service classes* containing many methods and code lines, whilst Smart Contracts code relies basically on the self contained code.

It is interesting to note the bell shaped behavior of the ABI metrics and of the Bytecode metric, which strongly differ from the shapes associated to lines of code or in general to other metrics. In the case of ABI this means that the amount of exposure of Smart Contracts to external interactions has a typical scale, provided by clear central values, even if the variance may be quite large. In other words Smart Contract exposure to the blockchain is very similar for most of the contracts, with no significative outliers, regardless the contract size in terms of LOC or other metrics. The bytecode displays a rather similar but less symmetric bell shape. In this case the behavior is clearly governed by the size constraints imposed by the costs of uploading very large Smart Contracts on the blockchain.

### 4.1 Analysing distributions of the metrics grouped by the pragma version

This section analyzes the distribution of some software metrics, such as the number of lines of code (LOC), the number of empty lines (Blanks), the number of functions (Functions) and the number of payable functions (payable), grouped by the pragma version. The pragma version is a directive which specifies how a compiler should process its input. The pragma version is not part of the grammar of a solidity programming language. The pragma version changes over time, as it is a way to identify the language used to categorize the states of solidity program language as it is developed and released. Smart Contracts should be annotated following this directive to avoid to be compiled by future compiler versions that might introduce incompatible changes. Despite this recommendation, not all smart contracts follow the pragma directive. The data set we consider in this paper consists of 85K of Smart Contracts and 19% of them did not follow the pragma directive. However, only the smart contracts following the pragma directive will be analysed to show a possible change or trend in how the smart contracts are developed over time.

For the following software metrics, functions, LOC and ABI, the peak of the distribution of smart contracts having the pragma version 0.5.* directives is shifted to the right compared to the smart contracts having the pragma version 0.4.* directives. As to what concerns the shape of the curves, the shape of the curve is broader in smart contracts having the pragma version 0.5.* directives, becoming progressively sharper with the decreasing of smart contracts having the pragma version 0.4.* directives.

### 4.2 Analysis of the number of contracts, libraries and interfaces

This section analyzes the number of Contracts, Libraries and Interfaces used in Smart Contracts written in solidity language during the time frame period from the year 2016 to the year 2021. Smart Contracts written in Solidity Program language consist of a number of contract declarations. Contracts in Solidity Program language are similar to classes in object-oriented programming (OOP) languages and, as in the case of OOP languages, there are four types of smart contracts: Abstract Contract, Interface Contract, Concrete Contract and Library Contract. In the following sections, the definition of each contract type will be provided and the use of these different contracts over the last 4 years will be analyzed.

#### 4.2.1 Abstract contract

Contracts are marked as Abstract Contracts when at least one of their functions lacks an implementation, as in the following example 1

**Listing 1**. Abstract Contract Example

35 // Abstract Contract

36 contract Notify

37 {

38  event Notified (address indexed _from, uint indexed _amount);

39  // functions signature

40  function notify (address _from, uint _amount) public returns (bool);

41 }

The functions that lack the implementation are named Abstract Functions. If a contract extends an Abstract Contract, it has to implement or define all the Abstract Functions of the extended Abstract Class, otherwise, it will be an Abstract Contract itself. Abstract contracts allow the use of patterns, such as the Template Method Design Pattern, and they allow to remove code duplication.

#### 4.2.2 Interfaces and libraries

Interface Contract was introduced in Solidity v0.4.11 on 3rd May 2017 [7]. An Interface Contract is similar to an Abstract Contract, but it cannot have any functions implemented. There are further restrictions such as it cannot inherit other Contracts or Interfaces.

Interface Contracts allow decoupling the definition of a contract from its implementation, providing better extensibility. In fact, when a Contract Interface is defined, the implementations of a new Contract can be provided for any existing functions without modifying their declarations. Interface Contracts are denoted by the interface keyword as in the following example 2

Listing 2. Interface Contract Example

42 // Interface Contract

43 interface Notify

44 {

45  event Notified(address indexed _from, uint indexed _amount);

46  // functions signature

47  function notify(address _from, uint _amount) public returns (bool);

48 }

A Concrete Contract has the implementation of all functions that are declared in the body of the contract. When a Concrete Contract implements an Interface Contract, it must provide the implementation of all the functions that are defined within the Interface implemented. If a contract extends an Abstract Contract, it needs to provide implementations for all functions not implemented in the extended Abstract Contract.

Library Contracts are similar to Concrete Contracts, but their purpose is different. A library is a type of contract that does not allow to use functions, such as Payable and Fallback, which provide a mechanism to collect or receive funds in Ethers. These limitations are enforced at compile-time, therefore making it impossible for a library to hold funds. A library is defined with the keyword library (library C {}) in the same way a contract is defined (contract A {}). Library Contracts are used to extract code away from the other Contracts for maintainability and reuse purposes.

**Fig 6:**
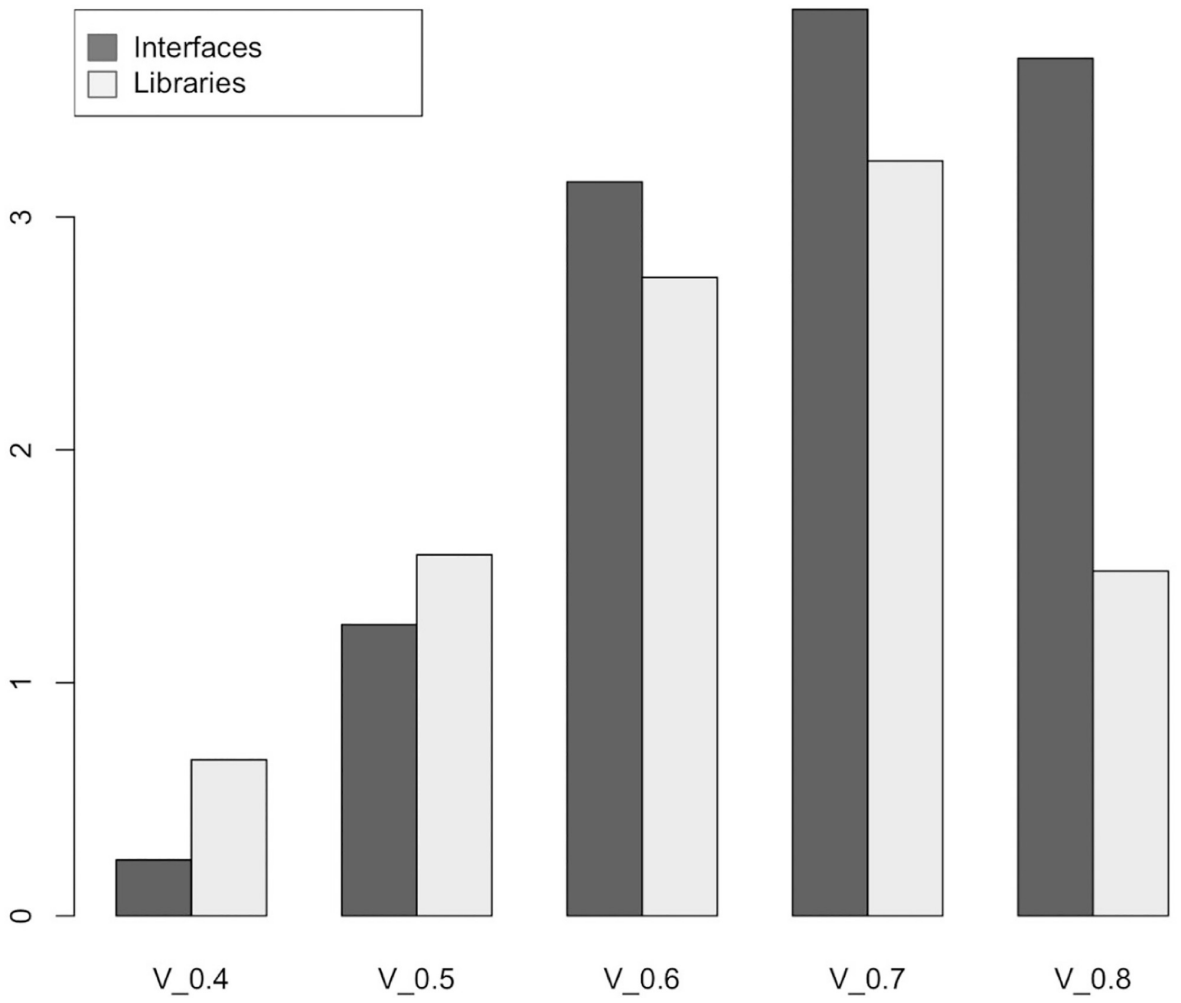
The average number of interfaces and libraries in Smart Contract.

**Fig 7:**
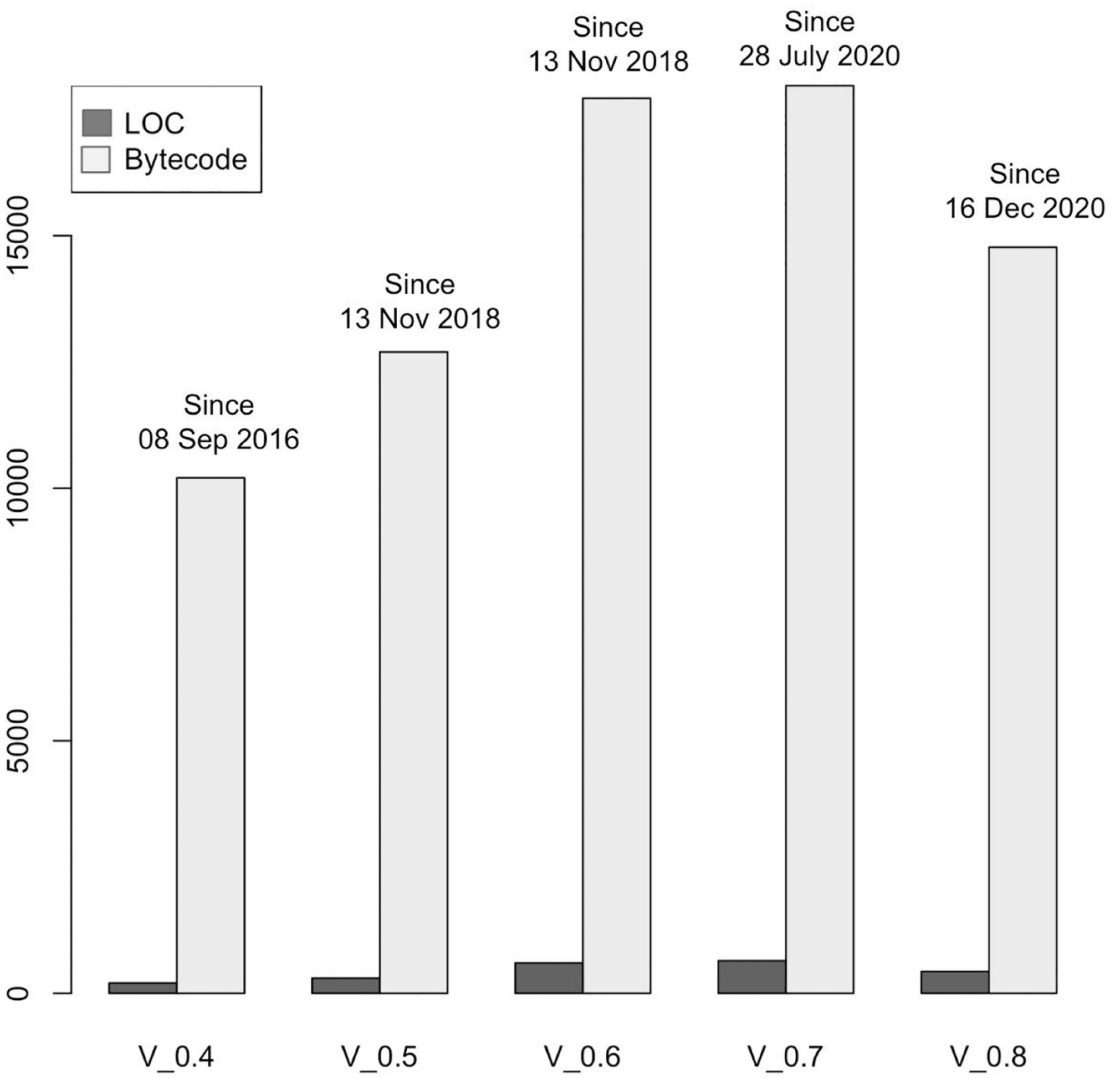
The average number of LOC and Bytecodes per Smart Contract.

Figs 6 and 7 show a growing trend in many software metrics such as the average number of LOC, Bytecode, number of interfaces, number of libraries, programming statements until the solidity version 0.7. Starting from solidity version v0.8 the trend is reversed. A plausible explanation for this trend can be found in the features’ changes of the Solidity programming language described in section 6.

**Fig 8:**
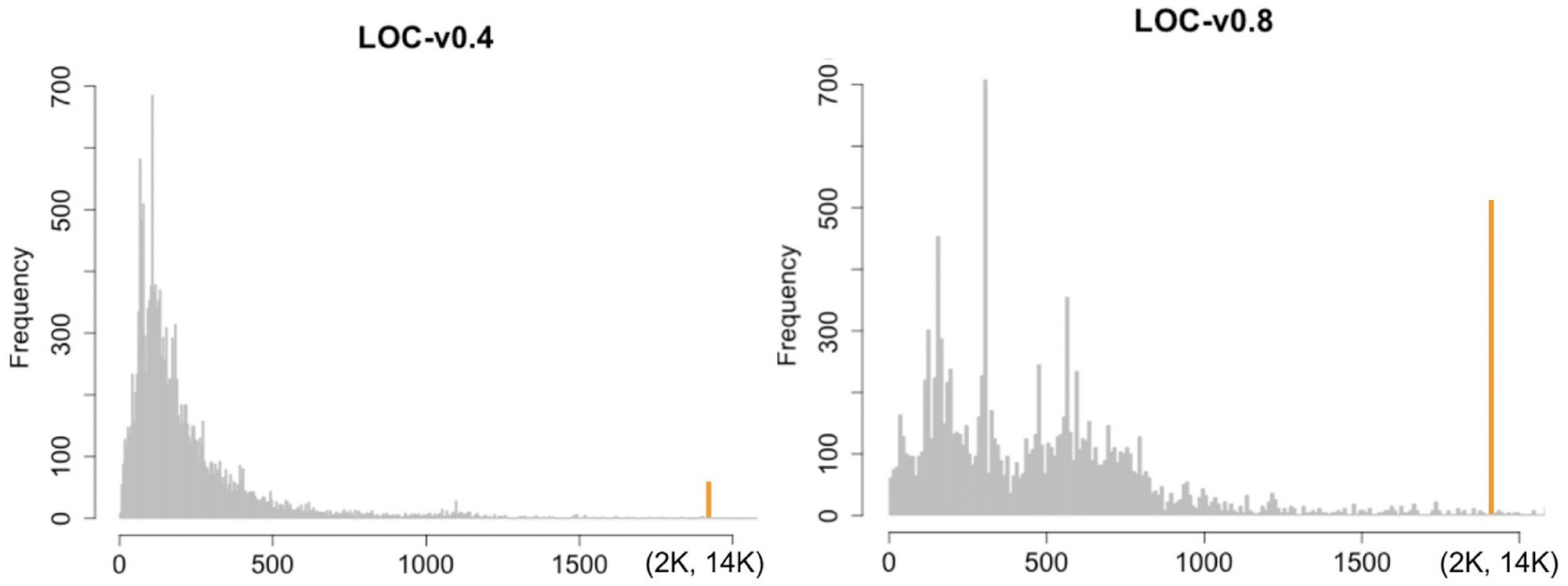
Smart Contracts’ LOC distribution vs. pragma version.

Fig 8 shows the frequency distribution of Lines of Code (LOC) for Smart Contract written respectively with Solidity version v0.4 (from 2016 to 2018) and Solidity v.0.8 (from 2020 onwards). Many Smart Contracts written before 2017 are in the LOC range from 0 to 500, and most of the Smart Contracts written after the 2020 year are in a larger LOC range between 0-1000. Moreover, the number of smart contracts having a LOC range between 4K-14K is one order of magnitude greater for smart contracts written after 2020.

#### 4.2.3 Replicated smart contracts

In this section we explain when and why we consider two Smart Contracts as different Smart Contracts. This is important for the aims of the paper because the results depend on the definition of replicated Smart Contracts. Some features of the Smart Contracts motivating the section are indeed the following ones:

- **Distinguishability**. Each Smart Contract in the Ethereum Blockchain is distinguishable from any other as it is identified by a unique address, i.e. a hash of 160 bits, and its code is stored on the blockchain. Smart Contracts can be deployed in the network by a user or by another Smart Contract or a cryptocurrency wallet. Each time a Smart Contract is deployed in the network, either in the main or in the test network, a unique address is associated with the Smart Contract even in the case the source code of two or more Smart Contracts is the same.
- **Immutability**. A user has no permission to change any Smart Contract deployed in the Blockchain. For example, if the user wants to correct a bug s/he is forced to redeploy the Smart Contract with a new unique address. As a result, on the blockchain there might be two or more almost identical Smart Contracts with different addresses. The fact that different addresses refer to the same Smart Contract lead us to suppose that many Smart Contracts might simply be “experiments” or contracts deployed in the blockchain to test and then modified according to the test results.
- **Inheritance**. The languages used to write Smart Contracts, such as Solidity, support multiple inheritance. When a Smart Contract inherits from multiple Smart Contracts, only a single Smart Contract is created on the blockchain, and the code from all the inherited Smart Contracts is copied into the new Smart Contract.

Based on these features, three ways to define the uniqueness of a smart contract will be outlined.

- Smart Contract A is different from a Smart Contract B because A and B have distinguishable addresses.
- Smart Contract A is different from a Smart Contract B if there is at least one different metric value.
- Smart Contract A is different from a Smart Contract B inheriting from the same Smart Contract C if the shared part of C does not overcome a given threshold, for example 80% of the code lines (LOC).

## 5 Statistical modeling

In order to get insights on the behavior of the statistical distributions underlying Smart Contracts software metrics we perform a best fitting analysis using a power law statistical distribution for best fitting the tails of the empirical distributions. Furthermore we performed a second analysis making use of the Log-normal statistical model. In fact, even when the power law model well represent the data in the tail it usually is unable to best fit the complete range of values in the statistical distributions.

To show the results of such analysis we don’t use histograms anymore, which are a rough approximation of a Probability Density Function (PDF).

Our methodology does not neglect any data and the use of cumulative complementary distributions allows to fully represent the statistical properties of the system analyzed (the blockchain software metrics in this specific case). This allows to model the system with analytical statistical distributions which provide more detailed and reliable information since all data points are included into the model.

The histogram representation in fact carries many drawbacks, in particular when data are power-law distributed in the tail. The problems with representing the empirical PDF are that it is sensitive to the binning of the histogram used to calculate the frequencies of occurrence, and that bins with very few elements are very sensitive to statistical noise. This causes a noisy spread of the points in the tail of the distribution, where the most interesting data lie. Furthermore, because of the binning, the information relative to each single data is lost. All these aspects make difficult to verify the power-law behavior in the tail. To overcome these problems from now on we systematically report the experimental CCDF (Complementary Cumulative Distribution Function) in log-log scale, as well as the best-fitting curves in many cases. This is convenient because, if the PDF (probability distribution function) has a power-law in the tail, the log-log plot displays a straight line for the raw data. This is a necessary but by no means a sufficient condition for power-law behavior. Thus we used log-log plots only for convenience of graphical representation, but all our calculations (CDF, CCDF, best fit procedures and the same analytical distribution functions we use) are always in normal scale. With this representation, there is no dependence on the binning, nor artificial statistical noise added to the tail of the data. If the PDF exhibits a power-law, so does the CCDF, with an exponent increased by one. Fitting the tail of the CCDF, or even the entire distribution, results in a major improvement in the quality of fit. An exhaustive discussion of all these issues may be found in [70]. This approach has already been proposed in literature to explain the power-law in the tail of various software properties [44, 52].

The CCDF is defined as 1 − *CDF*, where the CDF (Cumulative Distribution Function) is the integral of the PDF. Denoting by *p*(*x*) the probability distribution function, by *P*(*x*) the CDF, and by *G*(*x*) the CCDF, we have:

$$\begin{array}{c} G(x)=1-P(x) \end{array}$$ (1)

$$\begin{array}{c} P\left(x\right)=p\left(X\leq x\right)=\int_{-\infty }^{x}p\left(x^{\prime }\right)dx^{\prime } \end{array}$$ (2)

$$\begin{array}{c} G\left(x\right)=p\left(X\geq x\right)=\int_{x}^{\infty }p\left(x^{\prime }\right)dx^{\prime } \end{array}$$ (3)

The first distribution that we describe is the well-known Log-normal distribution. If we model a stochastic process in which new elements are introduced into the system units in amounts proportional to the actual number of the elements they contain, then the resulting element distribution is log-normal. All the units should have the same constant chance for being selected for the introduction of new elements [70]. This general scheme has been demonstrated to suit large software systems where, during software development, new classes are introduced into the system, and new dependencies –links– among them are created [52, 71]. The Log-normal has also been used to analyze the distribution of Lines of Code [72]. The Log-normal distribution has been also proposed in literature to explain different software properties ([52, 69, 73]).

Mathematically it is expressed by:

$$\begin{array}{c} p\left(x\right)=\frac{1}{\sqrt{2\pi \sigma }x}e^{-{\left(\frac{ln(x)-\mu }{2\sigma }\right)}^{2}} \end{array}$$ (4)

It exhibits a quasi-power-law behavior for a range of values, and provides high quality fits for data with power-law distribution with a final cut-off. Since in real data largest values are always limited and cannot actually tend to infinity, the log-normal is a very good candidate for fitting power-laws distributed data with a finite-size effect. Furthermore, it does not diverge for small values of the variable, and thus may also fit well the bulk of the distribution in the small values range.

The power-law is mathematically formulated as:

$$\begin{array}{c} p\left(x\right)≃x^{-\alpha } \end{array}$$ (5)

where *α* is the power-law exponent, the only parameter which characterizes the distribution, besides a normalization factor. Since for *α* ≥ 1 the function diverges in the origin, it cannot represent real data for its entire range of values. A lower cut-off, generally indicated *x*_0_, has to be introduced, and the power-law holds above *x*_0_. Thus, when fitting real data, this cut-off acts as a second parameter to be adjusted for best fitting purposes. Consequently, the data distribution is said to have a power-law in the tail, namely above *x*_0_.

**Fig 9:**
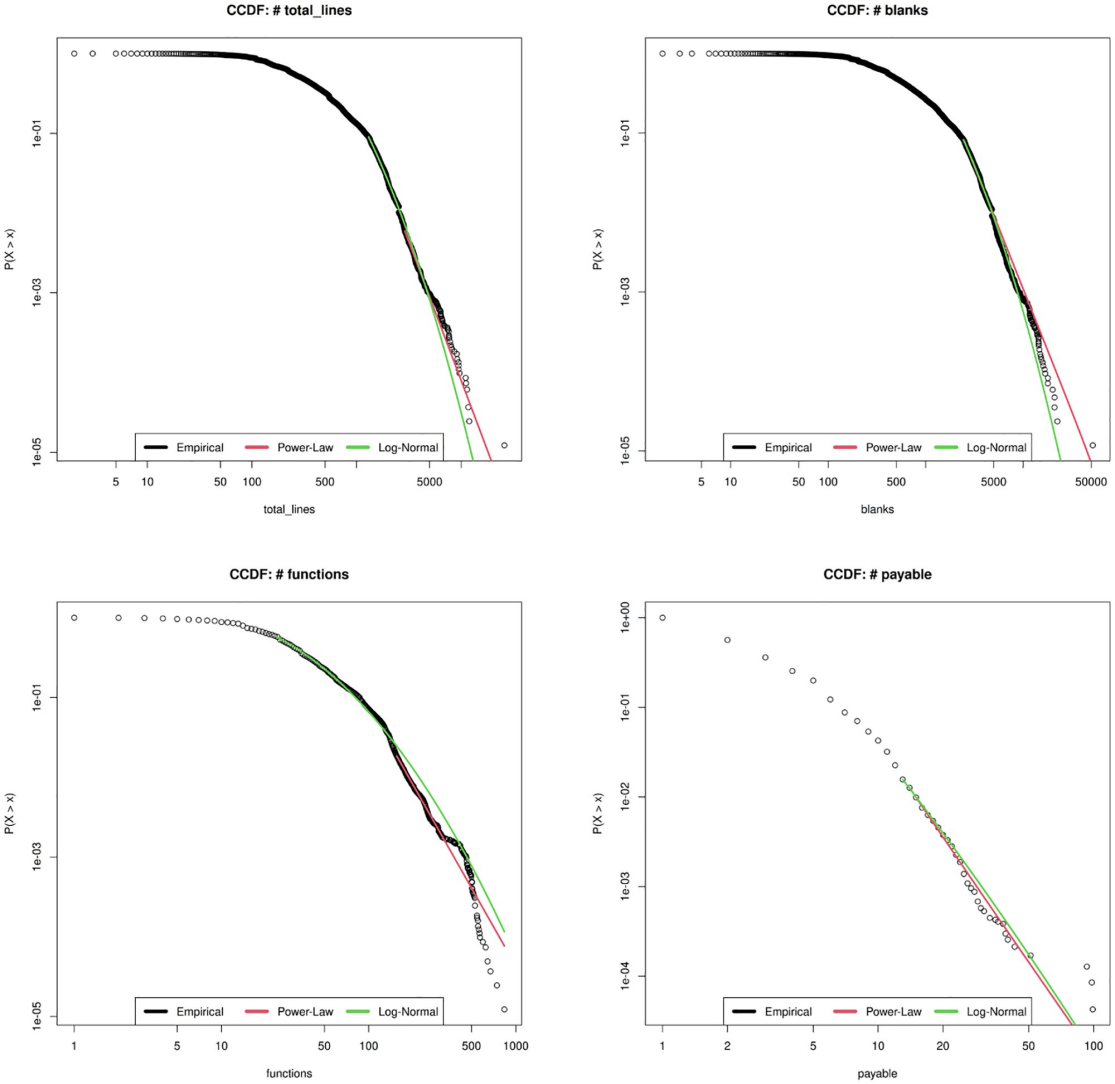
Power law and Log normal best fitting of the metrics Total lines, Blanks, Function and Payable.

**Fig 10:**
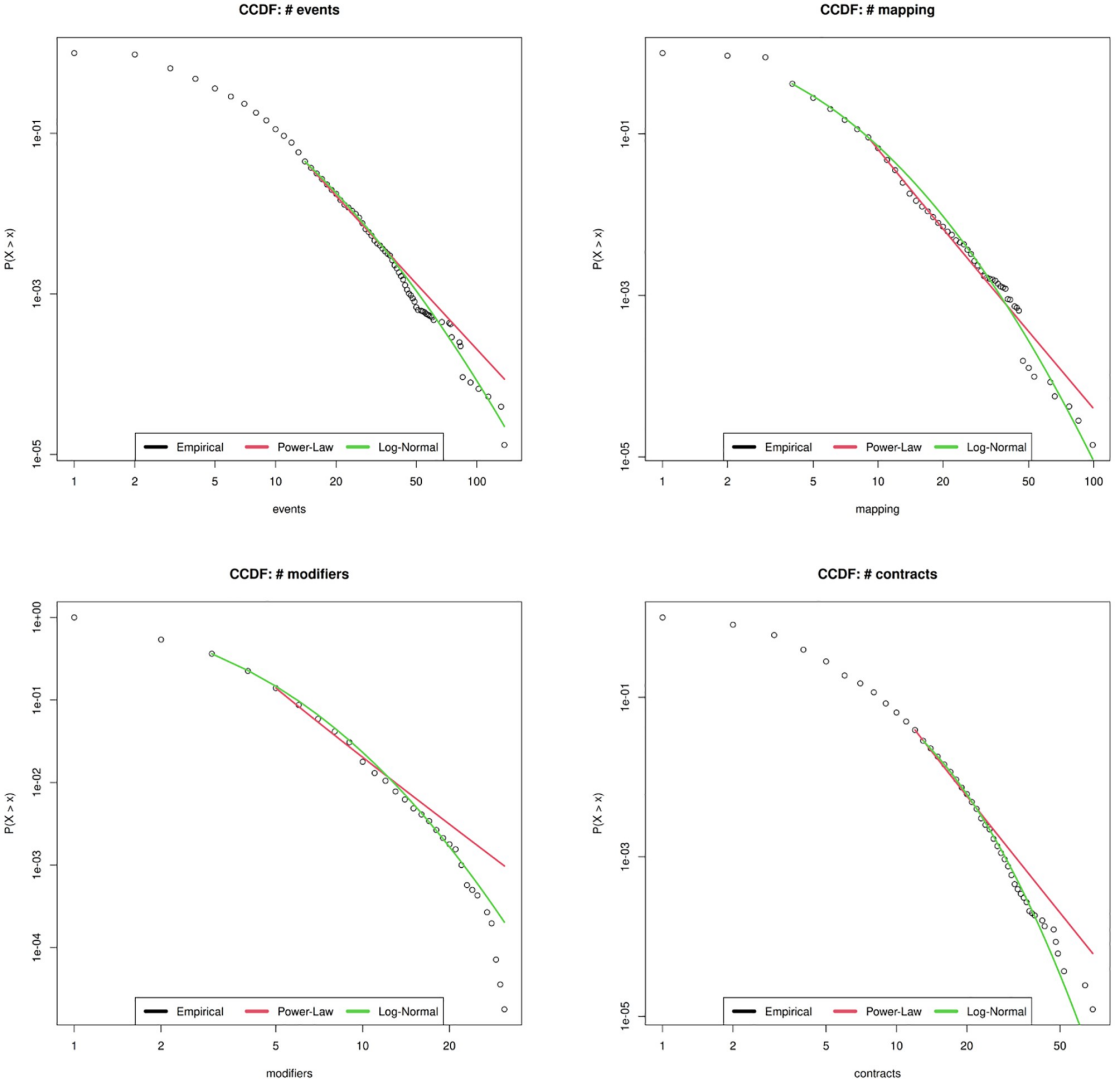
Power law and Log normal best fitting of the metrics Events, Mapping, Modifier and Contract.

In Fig 9 we show the best fitting plot for the power law model for the metrics Total lines, Blanks, Function, and Payable. The power law in the tail is clearly failed by all metrics. In Fig 10 Mapping and Modifier seems to follow a power law, confirmed also by the low values (D ≤ 0.05) of the Kolmogorof-Smirnov significance test value, but the range where the metrics behave according to a power law regime is too small.

**Fig 11:**
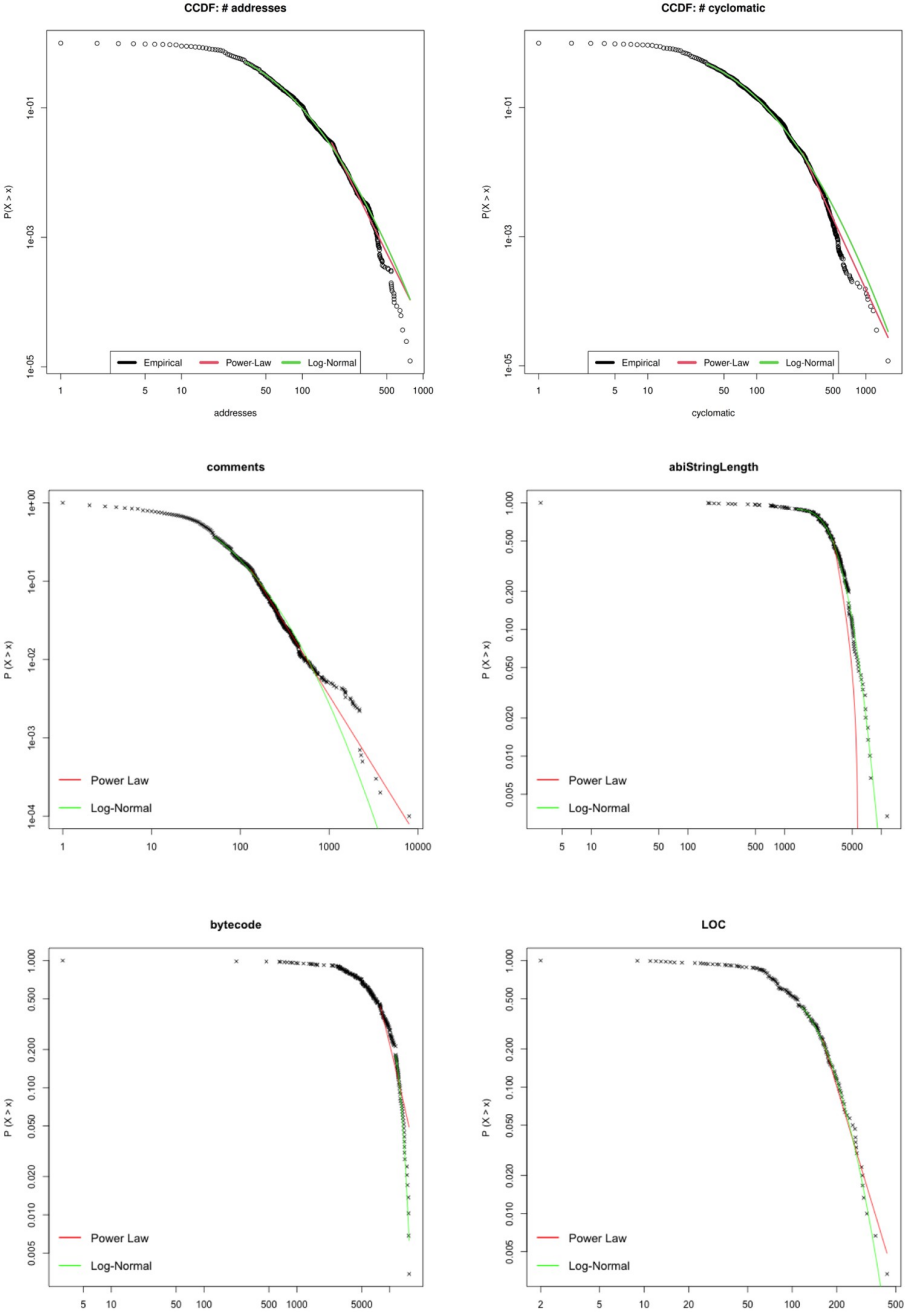
Power law and Log normal best fitting of the metrics Address, Cyclomatic, Comments, ABI, Bytecode and LOCS.

Fig 11 finally shows that a good candidate for a power law in the tail is the LOC metric, supported by a KS coefficient of significance of about 0.039. This suggests that also for the Smart Contract code the main size metric in software, the lines of code, shows properties similar to those of standard software systems. Also the Address metric displays a reasonable power law regime for a range of its values, showing a behaviour similar to that found for the metric “Name of Variables” in Java software [44]. Thus the usage of the keyword “Address” in Smart Contracts occurs in quantities which remind the usage of variable names in Java.

We then analyzed all the statistical distributions using a log-normal best fitting model.

In Fig 11 we show the Log-normal best fitting curves together with the empirical cumulative distribution functions for the Smart Contracts metrics Total lines, Blanks, Function and Payable. The first three metrics are nicely fitted by the Log-normal statistical distribution in the bulk, for low values of the metrics, but not in the tail, even if the *R*^2^ is quite close to one for each case (*R*^2^ ≥ 0.95). Such result confirms the previous one obtained for the power law model. The best fitting lacks mainly in the tail of the distribution, as expected. In fact the empirical distribution drops more rapidly than the best fitting curve because of the cut-off for large values of the metrics. This may be explained by the hypothesis that Smart Contract size metrics, like Total Lines of code, Functions and Blanks are upper bounded according to the size constraints associated to the deployment of Smart Contracts into the blockchain. The Payable metric results in a too poor statistic to be well fitted by a Log-normal distribution.

Fig 11 show the metrics Events, Mapping, Modifier and Contract. Mapping cannot be well fitted by a Log-normal, as it was very well explained by a power law in the range corresponding to the bulk of the distribution rather than in the tail. Also Events and Modifier do not suite a Lo-gnormal distribution and their *R*^2^ values are lower than 0.95. Finally Contract is quite well approximated in the bulk, but not in the tail, confirming once again the power law best fitting results.

Finally Fig 11 shows that the initial parts of Bytecode and ABI metrics well overlap with the Log-normal but as soon as the values crosses the central ones observed in the corresponding histograms the Log-normal curves tend to miss the empirical ones which drops quickly and do not display power law in the tail.

Address, Cyclomatic ad Comments rapidly drop with respect to the Log-normal model, even if the initial part presents some overlap with it. Again this may be ascribed to the upper bounds which limit the range of values reachable by these metrics. In particular Comments are less, on average, than in traditional software development. This is maybe due to the fact that Smart Contract software code is written with specific purpose and constraints, so that the same patterns are most likely found and do not need comment lines.

Finally the LOC metric is quite well represented by the Log-normal distribution both on the bulk and in the tail, and presents an *R*^2^ value larger than 0.98. This is quite in agreement with the results found in literature for the LOC metric in traditional software systems [44]. In some sense, this result is different from results obtained in similar studies, since it seems that this metric is not influenced by the peculiarity that can belong to Smart Contract software and tends to preserve the same statistical features found in traditional software systems.

Table 3 shows the final fitting parameters for the Power Law and Log-Normal distributions. We reported the *x*_*min*_ and *α* estimated parameters for the Power Law and *x*_*min*_, *log*(*μ*) and *log*(*σ*) estimated parameters for the Log-Normal.

**Table 3:** Fitting parameters for the power law and log-normal distributions. The *x*_*min*_ and *α* estimated parameters are reported for the Power Law. For the Log-Normal the *x*_*min*_, *log*(*μ*) and *log*(*σ*) estimated parameters are reported.

	Power Law			Log Normal				
Metric	*x*_*min*_	*α*	95% CI	*x*_*min*_	*log*(*μ*)	95% CI	*log*(*σ*)	95% CI
total lines	1323	3.33	3.327;3.341	150	5.75	5.748;5.758	1.105	1.104;1.108
blanks	308	2.94	2.925;2.949	23	3.97	3.972;3.984	1.032	1.029;1.033
functions	108	3.29	3.286;3.299	25	2.81	2.811;2.837	1.14	1.138;1.145
payable	5	3.01	2.994;3.021	1	0.29	0.296;0.312	1.16	1.155;1.160
events	11	3.29	3.282;3.295	3	1.08	1.071;1.084	0.965	0.963;0.967
mapping	3	2.92	2.915;2.935	4	0.28	0.26;0.31	1.06	1.064;1.076
modifiers	5	3.42	3.412;3.434	3	0.68	0.66;0.95	0.806	0.803;0.816
contracts	10	3.61	3.601;3.623	3	0.42	0.41;0.439	1.02	1.025;1.037
addresses	108	3.08	3.072;3.088	32	2.62	2.59;2.64	1.2	1.212;1.224
cyclomatic	161	3.15	3.145;3.159	36	3.68	3.675;3.698	1.04	1.041;1.049
comments	149	2.75	2.746;2.755	50	3.33	3.31;3.347	1.28	1.274;1.284
abi	174	3.1	3.095;3.155	3370	8.59	8.478;8.623	0.53	0.493;0.567
bytecode	11052	3.46	3.409;3.499	1830	9.02	8.993;9.032	0.65	0.642;0.661
LOC	148	2.62	2.574;2.642	161	0.38	-0.31;1.68	1.9	1.684;1.992

We validated our results using the bootstrap methodology in order to provide a 95% confidence interval for the estimated parameters. By default, the bootstrap function will use the Max Likelihood Estimator (MLE) to infer the parameter values and check all values of *x*_*min*_. The bootstrap procedure resamples the dataset with replacement for a large number of iterations (1000 in our case), for each iteration, all the parameter are estimated and at the end, a confidence interval is calculated. The bootstrap procedure provides more robust results.

In Table 3 we report the results of the bootstrap procedure, a 95% confidence intervals for the *α* parameter of the Power Law and *log*(*μ*) and *log*(*σ*) parameters of the Log-Normal is provided in the column next to each parameter.

## 6 Discussion

This section investigates the implications of the research based on the findings of our study. Some of the findings are the following:

- The Solidity program language has different styles of programming when compared to other high-level programming languages because of computational cost constraints and to be easier to understand for non-expert users.
- In the last two years the way of writing the smart contracts has been changing due to the the introduction new programme features in the last version of the compiler and because the Solidity developers started to implement more complex business logic over time.

As to what concerns the Solidity programming style, based on our findings (see Table 2), the number of iteration statements and conditional statements per line of code is respectively two and three orders of magnitude smaller than other high-level programming languages such as Java, C and python. Some relevant studies on this subject are [60, 73]. Furthermore authors in [74] show how cyclomatic complexity on Java code can reach very high values [74].

We assume that Smart Contract developers might have a tendency to minimize the use of branch statements (IF) and iterative statements (FOR, WHILE) because these instructions have a high computational cost when compared to other program statements such as the bitwise operations. Moreover, we assume that in order to increase public trust, the solidity developers tend to write smart contracts easy to understand. Indeed, a program easy to understand should have a low cyclomatic complexity although literature shows that readability, as intended by humans, weakly correlates with low cyclomatic metrics [75].

As far as the change in programming style, we observed at least two different distributions of software metrics data. First, many Smart Contracts written before 2017 are in the LOC range from 0 to 500, and most of the Smart Contracts written after 2020 year are in a larger LOC range between 0-1000. Moreover, the number of smart contracts written after 2020 and having a LOC in the outlier values (between 4K-14K) is one order of magnitude greater when compared to smart contracts written before 2017 and having a LOC in the same interval. The larger LOC range for Smart Contracts written after 2020 can be explained by the fact that the business logic of some Smart Contracts is deployed both 1) in longer source code and 2) in different Smart Contract addresses via specific pattern programs to bypass the source code size limit. Indeed, a Smart Contract has a code size limit equal to 24576 bytes and this limit was introduced to prevent denial-of-service (DOS) attacks. Originally, this limit was not a problem because the business logic of smart contracts was very simple as highlighted by our findings (LOC range from 0 to 500). However, in the last few years, the Solidity developers added more and more functionalities to their smart contracts until at some point they reached a code size limit. If the Solidity developers exceed this code size limit equal to 24576 bytes, they will not be allowed deploying the Smart Contract on the blockchain network. According to the grey literature, in the last few years, the Smart Contract size limit was overcome by using the “diamond pattern”. A “diamond Smart Contract” is a contract that gets its external functions from other contracts (called “facets”). On the contrary in traditional software power laws are commonly identified (eg. in Java programs) for general “size” metrics, defined for example in terms of the number of methods, constructors and other class features, where very large values of such metrics are commonly found [12].

Second, we observed a growing trend in many software metrics, such as the average number of LOC, Bytecode, number of interfaces, number of libraries, programming statements until the solidity version 0.7. Starting from Solidity version v0.8 the trend is reversed. A plausible explanation for this trend can be found in the changes of features in the Solidity programming language. The change of some features of the Solidity programming language is influencing the way Solidity software developers implement smart contracts from version 0.8 (released on 16 Dec 2020). Indeed, until Solidity version 0.7 (released on 28 July 2020), some characteristics of Solidity could lead many programming developers to introduce bugs in Smart Contracts. Fortunately, it was possible to mitigate the introduction of bugs by using external libraries such as OpenZepelling. For example, arithmetic operations in Solidity did not throw exceptions when an overflow occurred up to version 0.7 (the last release was on 16 Dec 2020). Indeed, this characteristic of Solidity can easily result in bugs, because programmers usually assume that a calculation that exceeds the memory space throws an error as in other high-level programming languages. Actually, starting from version 0.8, the Solidity compiler throws an exception when an overflow occurs in arithmetic operations. This means that the Solidity developers can update a Smart Contract or write a new Smart Contract via the newest compiler version without using external libraries, thus resulting in a Smart Contract smaller in size.

## 7 Conclusions

In this paper we studied Smart Contracts software metrics extracted from a data set of more than 85K Smart Contracts deployed on the Ethereum blockchain. We were interested in determining if, given the peculiarity related Smart Contract software development, the corresponding software metrics present differences in their statistical properties with respect to metrics extracted from traditional software systems and already largely studied in literature.

The assumptions are that resources are limited on the blockchain and such limitations may influence the way Smart Contracts are written. Our analysis dealt with source code metrics as well as with ABI and bytecode of Smart Contracts. Our main results show that, overall, the exposure of Smart Contracts to the interaction with the blockchain as qualitatively measured in terms of ABI size are quite similar to each other and there are not outliers Contracts. The distribution is compatible with a bell shaped statistical distribution where most of values tend to lie around a central value with some dispersion around it.

In general Smart Contracts metrics tend to suffer from blockchain limited resources constraints, since they tend to assume limited upper values. There is not the ubiquitous presence of fat tail distributions where there are values very far from the mean, even order of magnitude larger, as typical in traditional software. In Smart Contract software metrics large variations from the mean are substantially unknown and all the values are generally into a range of few standard deviations from the mean.

Finally the Smart Contract lines of code is the metric which more closely follow the statistical distribution of the corresponding metric in traditional software system and shows a truncated power law in the tail and an overall distribution which is well explained by a Log-normal distribution.
